# Initial Evidence for Positive Effects of a Psychological Preparation Program for MRI “iMReady” in Children with Neurofibromatosis Type I and Brain Tumors—How to Meet the Patients’ Needs Best

**DOI:** 10.3390/jcm12051902

**Published:** 2023-02-28

**Authors:** Liesa Josephine Weiler-Wichtl, Jonathan Fries, Verena Fohn-Erhold, Agathe Schwarzinger, Angelika Elisabeth Holzer, Thomas Pletschko, Julia Furtner-Srajer, Daniela Prayer, Paul Bär, Irene Slavc, Andreas Peyrl, Amedeo Azizi, Rita Hansl, Ulrike Leiss

**Affiliations:** 1 Department of Pediatrics and Adolescent Medicine, Medical University of Vienna, 1090 Vienna, Austria; 2 Department of Developmental and Educational Psychology, Faculty of Psychology, University of Vienna, 1010 Vienna, Austria; 3 Interdisciplinary Follow-Up Clinic for Childhood Cancer Survivors (IONA), Österreichische Gesundheitskasse (ÖGK), 1060 Vienna, Austria; 4 Department of Radiology and Nuclear Medicine, Medical University of Vienna, 1090 Vienna, Austria

**Keywords:** MRI training, neuro-oncology, psychosocial care, pediatric psycho-oncology, compliance, self-efficacy

## Abstract

To provide an effective alternative to sedation during MRI examinations in pediatric cancer and NF1 patients, the aims of the present study were to (1) exploratively evaluate a behavioral MRI training program, to (2) investigate potential moderators, as well as to (3) assess the patients’ well-being over the course of the intervention. A total of *n* = 87 patients of the neuro-oncology unit (mean age: 6.83 years) underwent a two-step MRI preparation program, including training inside the scanner, and were recorded using a process-oriented screening. In addition to the retrospective analysis of all data, a subset of 17 patients were also analyzed prospectively. Overall, 80% of the children receiving MRI preparation underwent the MRI scan without sedation, making the success rate almost five times higher than that of a group of 18 children that opted out of the training program. Memory, attentional difficulties, and hyperactivity were significant neuropsychological moderators for successful scanning. The training was associated with favorable psychological well-being. These findings suggest that our MRI preparation could present an alternative to sedation of young patients undergoing MRI examinations as well as a promising tool for improving patients’ treatment-related well-being.

## 1. Introduction

Magnetic resonance imaging (MRI) can be a demanding and stressful procedure for both children and their parents, requiring patients to lie motionless in a narrow, noisy tube [1,2]. Especially young children tend to have difficulties with self-restraint; the longer the procedure, the higher the risk for poor image quality due to motion artifacts, since the amount of patient movement within the scanner determines image quality and accuracy [3]. Possible strategies to reduce the risk of patient movement in young children are general anesthesia (GA) and sedation. In addition to requiring additional time, hospital bed and highly trained staff [4], sedation is also associated with rare, yet serious, well-documented adverse effects such as respiratory complications, aspiration, cardiac arrest, and vomiting [5,6].

A growing body of evidence supports an alternative approach: behavioral preparation and training methods show promising results in reducing the necessity of GA in young children [7]. Most studies have focused on how to increase compliance for non-sedated MRIs in various neurological and non-neurological patient groups (e.g., patients with diabetes, sickle-cell-disease, obsessive compulsive disorder) and healthy controls. However, very young children, children with developmental disorders, attention problems, or cognitive issues were often excluded in these samples. Specifically, only a few studies have included children with attention deficit hyperactivity disorder (ADHD), developmental delay, autism spectrum disorders (ASD), neurological impairment, anxiety, and other problems to lie still in the sample for the development and evaluation of preparation for MRI examinations [8,9,10]. The results illustrate the need for a differentiated consideration of predicting factors to increase compliance and to successfully comply with an MRI examination.

Cognitive and psychological issues are common and well-documented in pediatric brain tumor survivors and children with neurofibromatosis type 1 (NF-1), who are at an increased risk of developing a brain tumor. These issues include problems with attention, memory, processing speed, visuomotor function, learning difficulties, age related issues, as well as disease related stress factors (such as the recurrent experience of medical procedures) for patients and parents [11,12,13,14]. In brain tumor survivors, medical follow-up requires up to four regular MRI procedures per year [15]. The average duration of an MRI examination for these purposes is 30–60 min depending on which body parts need to be examined (brain MRI, brain and spinal cord MRI) [16,17]. Considering the frequency and duration of MRI examinations, as well as the specific challenges arising due to developmental, cognitive, or emotional issues, these children would considerably benefit from customized preparation programs that avoid the additional emotional and physical strain of GA [18,19,20].

The methods and aims of preceding studies on the topic are heterogenous, mostly focusing either on the reduction of sedation rates or on limiting movement artifacts to improve MRI image quality in pediatric patients, e.g., [10,21,22,23]. Preparations with a mock MRI, e.g., [7,24] or other techniques such as video animation [25,26], play therapy [26,27] or animal-assisted therapy [28] to prepare children for an MRI, as well as exposure to distracting techniques before [25] and during the MRI scan, e.g.,[23] have been demonstrated to effectively reduce the need for sedation. Furthermore, indirect effects have been reported, such as elevated sensitivity to the topic in the radiology department [25,29] and reduced waiting time for MRI due to fewer patients needing sedation/anesthesia [26]. However, little research has investigated the underlying methods of successful non-sedated MRI and the effects of MRI preparation programs on the psychological well-being of children undergoing scans. So far, temperament, medical experience, and parental expectations have been linked to higher MRI success [10]. Furthermore, some studies have focused on the reduction of anxiety and distress via appropriate MRI preparation [25,28,30].

Thus, the purpose of the present study was to retrospectively evaluate the effectiveness of MRI training “iMReady”, which aims to address the objectives discussed above, in addition to investigating the underlying mechanisms for non-sedated MRI success. Furthermore, the psychological effects of the training on its participants were analyzed prospectively to derive specific needs of a neuro-oncological patient group comprised of brain tumor and NF-1 patients.

## 2. Materials and Methods

### 2.1. Development and Content of the Training “iMReady”

The first version of the MRI training procedure “iMReady” was originally developed in 2008 and further adapted to become the standard of care at the local neuro-oncology unit. Its aim was to provide all children and adolescents with anticipatory guidance and developmentally appropriate preparatory information about the MRI, thereby implementing two standards formulated in 2015 in the Psychosocial Standards of Care Project for Childhood Cancer [18,20]. In line with the standards, the program addresses a wide variety of psychological concepts to decrease the psychosocial challenges caused by medical and diagnostic procedures and to increase compliance in MRI procedures overall, with or without GA. The incorporated concepts include psychoeducation, resource- and solution-oriented techniques, communication techniques, relaxation techniques, and reflection.

At the beginning of the training, every child receives an MRI training booklet containing important information about the MRI in a standardized, age-appropriate and visually appealing way. The booklet also provides practical material with enhanced stimulating elements to encourage the child to explore actively and multimodally, thereby contributing equally to the developmental and neuropsychological aspects of learning. Psychoeducational, active, practical, and reflective aspects are covered in two face-to-face training sessions. To reinforce the comprehension of the training process and to promote self-efficacy, the child is encouraged to bring the booklet to every session as well as to the diagnostic MRI.

**Figure 1:**
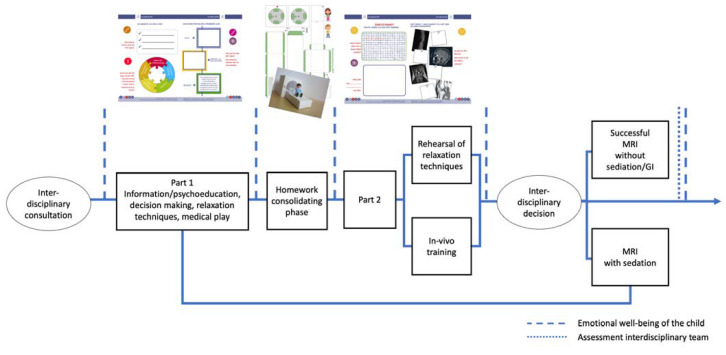
“iMReady” Workflow.

Considering age, developmental aspects, and interdisciplinary evaluation, a clinical psychologist or pediatrician may decide whether a patient qualifies for MRI training; children are referred to the training during a routine visit in the interdisciplinary outpatient clinic. Parents are informed about the purpose, conditions, and procedure of the training and encouraged to actively participate as co-therapists in the training to guarantee transfer and reinforce the effect of the training. The MRI training sessions are performed by an interdisciplinary team of medical doctors, radiologists, and nurses, but coordinated by a clinical psychologist trained to consider developmental and motivational aspects and possible cognitive, emotional, or social deficits in the participants. The training program consists of the following two sessions:

(1) The primary goal of the first session is the psychological and medical preparation, while also discussing the pros and cons about having the MRI without anesthesia. Hence, in addition to basic information and procedural knowledge about the MRI, instructions regardin appropriate clothing, metal objects, lying still, and the sound of the MRI are discussed with the participants. Role play techniques are used to build and practice coping strategies and action control. To further explore the topic, the children receive instructions for exercises, which they are asked to complete at home together with their parents. The homework is composed of two exercises: building a shoebox-sized paper MRI model (e.g., suitable for LEGO figurines) and continuing the role-play techniques with the paper model (Figure 1).
(2) In session two, the topics of the previous session are repeated before self-instruction and relaxation techniques for children are introduced and practiced. In general, it would be beneficial if MRI companies would build dummy machines for performing the training, in order not to occupy the scanning facilities. Considering available options, children and parents undergo a practice run in a 1.5 Tesla MRI (Siemens Aera) to build a relationship with MRI staff and to explore the MRI environment. During the practice run, children and parents can experience the MRI procedure and develop further helping strategies such as the use of additional blankets or pillows, a mirror on the head coil, or additional earplugs. The child has the opportunity to experience the room where the MRI takes place, the changing positions of the scanner bed, as well as the positions of parent, psychologist, and scanning administrator in a procedure that is convenient for them, involving age-appropriate communication methods. This includes methods for communication between the child and parent and/or team, which are developed individually (e.g., a hand signal from the child to the parent who is holding the hand or handset). One MRI sequence of approximately five minutes is performed, which gives children and parents the opportunity to experience the sound of the magnet and to make a first evaluation about picture quality. Finally, children, parents, and the psychologist reflect on the MRI training and together they decide whether the examination will be done with or without sedation. Children and parents are further instructed to regularly repeat and practice the acquired skills at home, while using relaxation, self-control, and operant techniques. More details on the proceedings can be found in Figure 1.

In 2017 the training was integrated into a quality improvement project (“Mein Logbuch—Ich kenne mich aus!”/“My Logbook—I know my way around!”) aiming to guide children throughout the entire disease trajectory (Trial registration identifier: NCT04474678). This is a method to apply the evidence-based S3 guideline for Psychosocial Care in Pediatric Oncology and Hematology [31], using a multi-stage process of Plan-Do-Study-Act (PDSA) cycles [32], including a Delphi survey to incorporate the expertise of psychosocial workers in the German-speaking region. Results emphasize the necessity of standardized psychological support to enable an evaluation and optimization of psychosocial care. This paper relies on adopting the patients’ perspective to evaluate the patients’ emotional well-being and their subjective sense of expertise (evaluated by medical staff and the patients themselves) [33]. The resulting tool consists of various booklets, each focusing on one topic and the corresponding psychosocial stressors encountered by patients during cancer treatment (e.g., “ABC of chemotherapy”, “Mission stem cell transplantation”), including “iMReady” as crucial examination [33].

### 2.2. Ethical Approval

This study was performed in line with the principles of the Declaration of Helsinki. Approval was granted by the local Ethics Committee of the Medical University of Vienna.

### 2.3. Participants

The total study sample was composed of 105 patients with a mean age of 6.83 years (*SD* = 2.04; range = 4 to 14). All children aged four or older that were admitted to the facility where this program was developed were invited to participate in the training session. They were given the voluntary option to participate in the ongoing explorative study to evaluate the training’s effectiveness. Since we expected that all children could benefit from this training program, no specific exclusion criteria were applied.

For the 53 female participants (50.5%), the mean age was 6.21 (*SD* = 1.64). For male participants it was 7.46 (*SD* = 2.23). All in all, 37 children attended pre-school or kindergarten. The 52 children and adolescents who attended school were on average in second grade (median). For the 16 remaining children, no education information was available. The majority of participants spoke German as their first language (66%). For children who did not speak German, non-verbal methods were used and, if possible, parents helped in translation.

Most of the patients (72.69%) suffered from brain tumors. The mean onset age of the medical condition was at 3.01 years (*SD* = 2.76). Thirty-three patients suffered from NF1 in the absence of brain tumors. There was a certain degree of overlap between the NF1 and the tumor group. Twenty-one of the patients afflicted by NF1 also exhibited brain tumor diagnoses. More information on sample characteristics as well as the patients´ medical details can be found in Table 1.

Of all the participants enrolled in the program, 87 (83%) received MRI training, while 18 (17%) did not, due to scheduling reasons (*n* = 9) or because the parents did not want their children to receive the intervention (*n* = 8); for one child, the reason was unknown. Although the current study did not recruit a matched control group, we used this group of non-participating children as a reference group. This group will henceforth be referred to as “dropout group”. The dropout group was composed of 8 girls (44%) and 10 boys (56%). The mean age in the dropout group was 7 years (*SD* = 2.33). In terms of diagnosis, five children suffered from NF-1 (28%), while 13 (72%) suffered from a brain tumor in the absence of NF-1.

As part of the evidence- and consensus-based development of the training, the updated version was deployed in the fall of 2018. This second phase involved a longitudinal assessment of emotional well-being. Of 17 patients participating, 10 were female (59%). The mean age was 5.65 years (*SD* = 1.37, range = 4 to 9 years). Twelve children spoke German as their first language (71%). Thirteen patients suffered from brain tumors (76%), while 4 did not (24%). Eight participants suffered from NF1; 4 of these also had brain tumors. All patients with brain tumors received oncological treatment according to protocol. Participants in the prospective analysis were a subsample of the total sample (*n* = 105). These children underwent the same procedure as the remainder of the sample; however, the emotional assessment was exclusively carried out in this subsample.

### 2.4. Design

#### 2.4.1. Retrospective Analysis

In this explorative, observational study, all MRI training referrals between January 2014 and October 2018 were retrospectively investigated for training effectiveness and underlying medical, neuropsychological, or sociodemographic moderators of MRI success. MRI training was labeled successful when patients could lie still for a minimum of one MRI sequence. An MRI scan was labeled successful when it yielded interpretable results (with none to moderate movement artifacts) as defined by a neuroradiologist, and which did not require the use of sedation/anesthesia. The quality of MRI pictures was rated on a three-point scale (no or mild motion artifacts; moderate motion artifacts; severe motion artifacts or termination). As part of the standard of care protocol at the neuro-oncology unit, all patients were administered a comprehensive neuropsychological test battery before or after the MRI training. For this retrospective study, the different pre-existing neuropsychological test results relevant to the study question were included in the analysis. Details on the neuropsychological tests administered can be found in Table 2.

#### 2.4.2. Prospective Analysis

The prospective analysis followed an explorative approach as well. In this second part of the current research, we focused on the patients’ perceived well-being, knowledge about the procedure of the MRI examination, and how to cope with the stress potentially caused by it. To this end, we assessed the patients’ emotions using an intuitive, visual approach. The evaluation of patients’ emotional well-being was carried out using an array of images representing emotional states [44]. The participating children were asked to choose three of the 18 presented images that best described their emotional well-being throughout the MRI training. For subsequent analysis, these emotional displays were categorized into positive, neutral, and negative emotions. The evaluation of emotional well-being was conducted longitudinally over five different time points—before and after session one, before and after session 2, and after the diagnostic MRI.

The evaluation of the degree of confidence and knowledge of the children and their parents was carried out by medical staff in the radiology department during the diagnostic MRI scan. Due to procedural constraints, data were only available for children that completed their MRI without general anesthesia. Medical staff scored each category (child well-informed, child secure, accompanying person well-informed, accompanying person secure) on a scale from 1 to 10.

### 2.5. Statistical Analysis

For data analysis, a combination of descriptive statistics and statistical inference was used. The sample was described using common statistical indicators. If the level of measurement allowed for it, a Welch’s *t*-test was applied due to its robustness with unequal variances and sample sizes. This test generates non-discrete degrees of freedom [45]. Categorical variables were analyzed using Chi-squared-tests of independence. Where possible, measures of effect size were computed. In two-group comparisons, Cohen’s *d* was calculated. For contingency tables with polytomous categorical variables, Cramér’s *V* was calculated, whereby an odds ratio for 2 × 2 contingency tables was adopted.

The patients in this intervention reported their emotional state at five different points of time over the course of the study by selecting three faces out of a range of emotional displays. Subsequently, these emotions were categorized into positive, neutral, and negative emotions. Since the data was categorical and non-independent due to the longitudinal design, general linear model techniques were not applicable. Therefore, the change of selected emotions over the course of the intervention was analyzed using generalized linear mixed models (GLMM) with Poisson distribution and log link function. Maximum likelihood with Laplace approximation was used as an estimation method. These models are extensions of the general linear model which have been adapted to account for the categorical and dependent nature of the data [46].

A type I error rate of 0.05 was chosen in all analyses, thus tests that yielded probabilities below that level were considered statistically significant. No adjustments for type I error inflation were made; instead, effect sizes were reported [47]. In all analyses, the statistical programming environment R (version 3.60 for Mac OS) was applied; for GLMM analyses, the R package lme4 was used (version 1.1-24); graphics were created using the R package ggplot2 (version 3.4.1).

## 3. Results

### 3.1. Effect of MRI Training (Retrospective Analysis)

**Figure 2:**
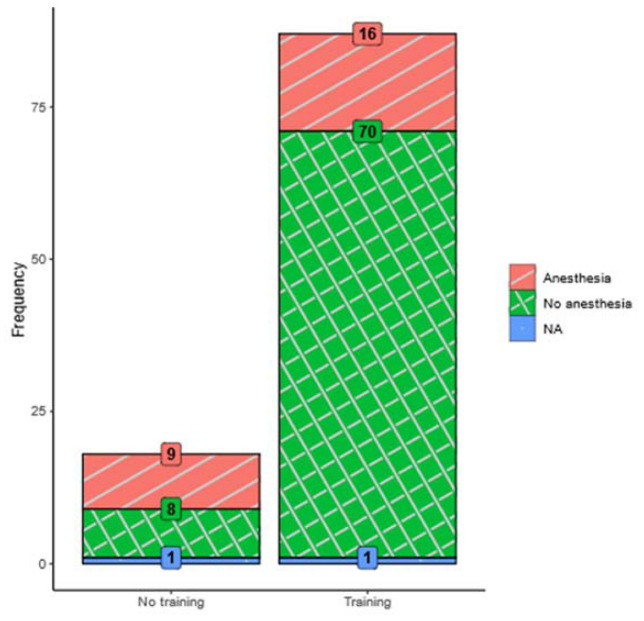
Stacked barplot for MRI success rates in both the intervention and the non-intervention group.

The principal goal of this intervention was to empower children and adolescents to manage undergoing an MRI without the need for general anesthesia. The term MRI success will be used for MRI performance without general anesthesia. All patients attended MRI training. The overall success rate for the total sample of 105 patients was 74%. The group that participated in the training program (*n* = 87) performed significantly better (80% success rate, 70 out of 87) compared to the group (*n* = 18) that did not receive training (44% success rate, 8 out of 18; χ^2^ = 9.1, *df* = 1, *p* < 0.01). The odds ratio indicates that the chance of successfully managing an MRI without anesthesia was almost five times as high in the group that received MRI training compared to the group that did not (*OR* = 4.92, 95% *CI* = 1.64; 14.73, *p* = <0.01). Moreover, MRI picture quality was acceptable in the intervention group. See Figure 2 for a visual comparison of MRI success rates per group.

### 3.2. Moderators of MRI Success (Retrospective Analysis)

#### 3.2.1. Sociodemographic and Medical Associations

There was no sex difference in the probability of MRI success (*χ*^2^ = 0.78, *df* = 1, *p* = 0.28, *OR* = 0.66). Age (*t* = 0.33, *df* = 47.18, *p* = 0.74, Cohen’s *d* = 0.07) and grade (*t* = −1.06, *df* = 33.33, *p* = 0.30, Cohen’s *d* = −0.21) did not differ among patients that managed their scans without general anesthesia and those who required anesthesia. There was no significant association between the type of education and MRI success (*χ*^2^ = 0.00, *df* = 1, *p* = 0.95, *OR* = 0.95), i.e., the rate of need for sedation during scans did not vary between children in special education compared to patients in regular schools. Notably, patients’ first languages were not associated with MRI success, indicating that non-German native speakers did not have more difficulties than German native speakers (*χ*^2^ = 0.02, *df* = 1, *p* = 0.90, *OR* = 1.06).

MRI success rates in patients suffering from NF1 (without a brain tumor) were compared with patients suffering from a brain tumor; there was no significant association between the two variables (*χ*^2^ = 1.84, *df* = 1, *p* = 0.17, *OR* = 1.9). Furthermore, the MRI success rates between different treatment options were categorized into four tiers: observance only, surgery only, surgery and chemo-, radio- or antiangiogenic therapy, chemo-, radio- or antiangiogenic therapy. Subsequent comparison showed no significant association between the treatment arm and MRI success (*χ*^2^ = 3.62, *df* = 3, *p* = 0.30, Cramér’s *V* = 0.19).

However, the onset age of the diagnosed medical condition differed significantly between patients that managed the MRI without anesthesia and those that required anesthesia (*t* = −2.17, *df* = 56.69, *p* = 0.03, Cohen’s *d* = −0.44). Patients that required anesthesia exhibited a significantly lower mean age (2.21 vs. 3.1 years, respectively) at the onset of their medical conditions.

#### 3.2.2. Neuropsychological and Behavioral Associations

**Figure 3:**
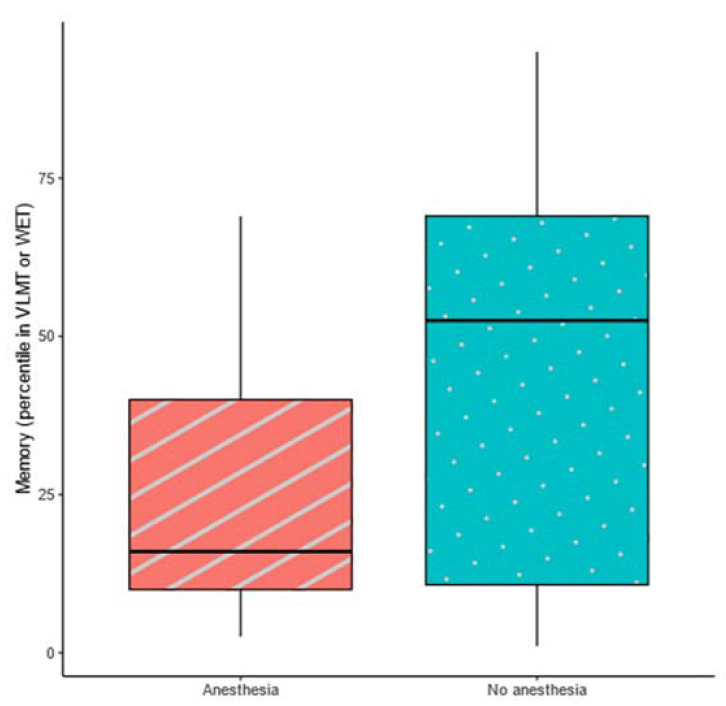
Boxplot of patients’ memory, as measured by VLMT or WET, compared by MRI success.

The successful completion of their MRI scans without requiring general anesthesia could not be explained by IQ, attention, or concentration. However, memory (VLMT [40] or WET [41]) showed a significant, medium effect size which is visualized in a boxplot in Figure 3. See Table 2 for the tests used and Table 3 for more detailed information on neuropsychological data. Patients that needed anesthesia scored significantly lower in their memory tests (*M* = 25.91, *SD* = 22.44) compared to patients that did not need general anesthesia (*M* = 46.33, *SD* = 31.12).

To test for associations between MRI success and behavioral or emotional symptoms (SDQ) [43], the scores were dichotomized into the categories normal and critical range. The only category that showed a significant association with MRI success was hyperactivity and attentional difficulties. Forty-eight percent of patients that required anesthesia during the MRI exhibited high or very high levels of hyperactivity and attentional difficulties, whereas only 19 percent of patients that did not require anesthesia exhibited high or very high levels (Table 3).

The dropout group did not differ significantly from the intervention group regarding memory (*t* = 0.82, *df* = 20.25, *p* = 0.53, *d* = 0.22) or their scores in SDQ hyperactivity and attentional difficulties (*χ*^2^ = 2.21, *df* = 3, *p* = 0.53, Cramér’s *V* = 0.60).

#### 3.2.3. Evaluation of Patients’ Emotional Well-Being (Prospective Analysis)

**Figure 4:**
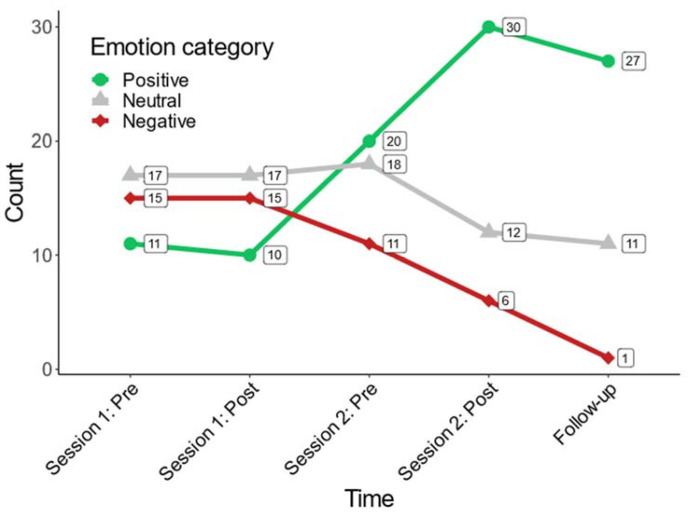
Line plot of frequencies of selected emotions over time. Note, Labels correspond to frequencies for the respective emotions at each time of measurement.

We analyzed the frequencies of positive, neutral, and negative emotions at each point of the prospective study. Figure 4. shows the frequency at which patients selected each category over the course of the study. Separate GLMM’s were applied for positive, neutral, and negative emotions. The point of time was included as a fixed-effect variable, while participant ID was included as a random-effect variable to account for the dependent data structure. Over the course of the five points of measurement across the study, negative emotions dropped significantly (*beta* = −0.41, *z* = −3.64, *p* < 0.01, *R*^2^_marginal_ = 0.12), indicating a reduction of 0.41 in the count of negative emotions reported by participants with every point of measurement over the course of the study. Meanwhile, positive emotions were selected more frequently (*beta* = 0.27, *z* = −2.68, *p* = 0.00, *R*^2^_marginal_ = 0.14), indicating that participants reported 0.27 positive emotions more with every point of measurement. Neutral emotions showed a moderate decline which did not reach statistical significance (*beta* = −0.11, *z* = −1.38, *p* = 0.17, *R*^2^_marginal_ = 0.02). In these models, *R*^2^_marginal_ signifies the amount of variance explained by change over the course of the intervention.

#### 3.2.4. Interdisciplinary Evaluation (Prospective Analysis)

All categories evaluated by the medical staff exhibited median values above “8” on a visual analog scale (0–10), indicating generally favorable values. “Child secure” exhibited the lowest median as well as the largest variance (*M* = 8.74, *SD* = 1.34), with an overall high information level (“child well-informed” *M* = 9.25, *SD* = 0.73) The highest medians and the smallest variances were found in the categories concerning the accompanying persons, “Accompanying person well-informed/secure” (*M* = 9.42/9.42, *SD* = 0.51/0.52).

## 4. Discussion

In this exploratory, observational study, our primary goal was to demonstrate a novel training procedure designed to prepare children for MRI exams. Although the design of the current study does not allow for causal inference, these initial results suggest that the children who participated in the program were more likely to exhibit desirable outcomes in MRI exams. Furthermore, our results suggest that over the course of the intervention, negative emotionality declined, while positive emotionality increased.

Children who received prior training exhibited a five times higher chance of having a successful diagnostic MRI scan than children who did not receive a training. Furthermore, 81.4% of children who received the MRI training managed the diagnostic MRI scan with good image quality, while children who did not receive the training had only a 47% chance of a successful MRI. Considering the high burden of disease and the great frequency at which childhood cancer patients must undergo investigations, this chance of reducing the number of interventions and sedations could help reduce psychological and physiological stress in patients and their families.

The encountered success rate is comparable to success rates in children with developmental delay and ADHD [10] but slightly lower than in prior studies with different patient populations [21,24,41]. The lower success rates in children who suffer from brain tumors or neurofibromatosis compared to other patient groups could be explained by the high mental stress faced by families when checking for treatment success or possible tumor recurrence, as well as cognitive issues often faced in these patient groups, e.g., [13,14]. Another possible explanation could address differences in the duration of MRI scans among the study samples [10] ranging from 7 [29] up to 100 min [24]. Hence, randomized control trials need to be carried out to enable a more valid and well-founded judgement of the benefits of this novel program. Since the overall image quality was judged to be satisfactory in the entire group, it appears that for the future, image quality alone will not be the essential outcome criterion for the successful completion of an MRI training. From a clinical perspective, it seems even more appropriate to focus on the experienced well-being as well as the level of information as outcome criteria, which both contribute significantly to higher compliance and even more to a resilient outcome in life-long disease management [8,24,48,49].

Only a small number of studies have investigated the mechanisms underlying successful MRI scans in children showing associations with cognitive and language skills, [22] age [50], temperament, patients’ knowledge of the procedure, and previous medical experience [10]. Interestingly, in the present study neither age, gender, treatment arm, nor the mother tongue had an influence on success rates. However, neuropsychological risk factors for MRI success are low scores in long-term memory, as well as reported attentional problems, two cognitive impairments that pediatric brain tumor and NF1 patients are especially vulnerable to [51]. Moreover, difficulties regarding memory and attention are often related to genetic components and early disease onset [52,53], which in turn was associated with a reduced MRI success rate. This is clinically plausible, since, for example, memory deficits do not only make it more difficult to remember the content of the training and particularly strategies, to lie still and to cope with stressful situations, but can also lead to an increased feeling of insecurity, which might be multiplied in stress evoking situations such as MRI scans [54]. Overall, the neuropsychological moderators clearly show that it is important to customize the training to the patient’s neuropsychological outcome and developmental stage, which in turn need highly specialized and well-trained psychological staff. Further research is necessary to evaluate if prior memory or attentional training leads to an even higher success rate.

The value of integrating the parents’ perspective is further underlined by the fact that only attentional difficulties based on external observation by the parents modified MRI success while results in tests on attention and concentration did not. However, this might be due to the nature of the tests chosen to check for attentional difficulties. In addition to the evaluation of focused attention (KHV-VK and KITAP—distractibility condition), future studies should include an assessment of other dimensions of attention as well as record the exact duration of the individuals’ sessions to allow for an investigation of a possible relationship between intervention time and the program’s effectiveness, to derive practical implications for clinical assessment.

Compared to other MRI training tools, the major difference of the present approach lies in the integration of role play techniques as well as in vivo training, two learning strategies that are especially effective for elaborating and solidifying the training content [27,55]. In a preventive sense, strategies considering emotional outcome (e.g., reduction and/or avoidance of fears) are implemented at an early stage to ensure short-term and long-term follow-up care [16,23,49]. The results emphasize the importance of the training being performed by clinical psychologists specializing in child development and neuropsychology who closely work together with medical doctors and radiotechnology assistants in an integrated care system [26,56,57]. In the future it would be of great interest to adopt the assessment scale of the interdisciplinary report to get more detailed information on the performance during MRI to derive further practical implications for psychosocial support. Transferring the training methods into daily life can additionally help to solidify the learned content, which was supported in the evaluated program by the parents acting as co-therapists. Additionally, in the aim of increasing immediate treatment success, the close interaction with and evaluation of the patients by this comprehensive team can also enable the professionals to better judge whether patients are prepared to undergo MRI examinations without sedation. In turn, the risk of stress and trauma can be reduced [10,17]. Hence, the present study was the first to show that children experience a variety of emotions with regard to an MRI, which might change over time. While prior work has exclusively focused on fear, anxiety, and distress [8,22,23,28,30] the use of an array of 18 different emotional displays categorized into positive, neutral, and negative, allowed for a more detailed evaluation of patients’ feelings in relation to the MRI procedure. The results illustrate a high variability of emotional experience and highlight positive emotions associated with dealing with an MRI examination. Tracking the emotional state over five time points, a decrease in negative and an increase in positive emotions was evident, the latter reaching a peak after the completion of the diagnostic MRI, which might be related to relief after the completion of the diagnostic procedure. The changes in emotional wellbeing (a reduction followed by an increase in positive emotions, only with continuation of the training), illustrate the shifting process in the therapeutic procedure: information transfer (disengagement—reduction of positive emotions); learning new strategies in dealing (shift—reduction of positive emotions); integration into everyday life (engage—stabilization or increase of positive emotions) [17]. The results highlight the fact that simple information is not sufficient. Moreover, standing alone without follow-up appointments offering coping strategies can thus have the opposite effect and increase uncertainty.

The underlying aim of the current study was to systematically visualize experiences from clinical practice in terms of outreach opportunities, outcome in well-being, and evidence of perceived levels of information as important aspects of health literacy and resilient coping with necessary medical procedures. Furthermore, we assume that these results will provide a basis for further research in the context of controlled randomized studies. This may reveal more insight concerning the effects of the individual training aspects as well as the actual strategies used to successfully complete an MRI examination (both in terms of image quality, but also in terms of a positive processing of the situation).

## 5. Limitations and Future Directions

Significantly, the current study was explorative in nature. This is reflected by specific aspects of the research. First, hypotheses of the study were not pre-registered, thus results of all analyses are not to be interpreted as confirmatory hypothesis tests but as preliminary findings. Moreover, to estimate the effectiveness of the “iMReady” program, we employed a group of children that did not take part in the intervention as reference group (i.e., the dropout group). These children cannot be considered a matched control group because they opted out of the “iMReady” program for various reasons that were not controlled by the researchers and may have introduced systematic bias into the comparisons. Thus, the findings of intervention to the dropout group comparisons must be interpreted as explorative effect estimates. Future research carried out in the form of controlled trials will reveal if these effects are robust and reproducible.

Emotional well-being was only assessed with a limited number of patients during the latter part of the study for which no reference group was available. Therefore, although the findings are promising, the decline in negative emotions and simultaneous increase in positive emotions cannot be causally attributed to the MRI preparation program. Future endeavors should evaluate whether the observed treatment effect also emerges within a controlled design.

The sustainability of stress relieving strategies and coping mechanisms promoted through MRI training is an interesting field for further research. We would expect, among other things, that children with NF1 need more support in preparation, even though this has not been shown in the data. A differentiated evaluation with respect to disease and tumor entities is particularly desirable to allow for more specific statements regarding selected risk groups.

## 6. Conclusions

The MRI Training “iMReady” is a highly feasible, evidence-based tool, which can easily be adjusted to the special needs of the heterogenous patient group in pediatric neuro-oncology. The results of the present study suggest that treatment compliance is moderated by disease-related cognitive, physical, and behavioral difficulties. Tailored trainings, such as the one demonstrated here, may be a promising strategy in counteracting compliance problems and increasing MRI performance. Furthermore, it was the first study to evaluate patients’ emotional experiences related to MRI procedures, indicating a substantial improvement in patients’ self-reported emotional well-being throughout the psychoeducational program. Further studies with larger sample sizes as well as a randomly assigned, matched control group are necessary to more thoroughly investigate the patient characteristics that are most relevant to prediction of successful treatment, to allow for the trainings to be tailored to individual needs. In addition, to ensure implementation and to avoid competition between training time slots (20–30 min) and actual MRI examinations (amounting to increased waiting time), it would be beneficial if the MRI companies would build dummy machines with which to perform the training.

## Figures and Tables

**Table 1:** Descriptive date (Sociodemographic and medical details).

Variable	Frequency (%)
Sex	
Female	53 (50.5%)
Male	52 (49.5%)
First language	
German	60 (66%)
Turkish	5 (6%)
Serbian	3 (4%)
Bosnian	1 (1%)
Polish	1 (1%)
Other	9 (10%)
Bilingual	12 (13%)
Education	
Kindergarten/pre-school	38 (36%)
Primary school	44 (41%)
High school	8 (8%)
Not available	16 (15%)
Special education needs	
Regular school	34 (32%)
Special education	12 (12%)
Not available	59 (56%)
Diagnosis	
NF1	33 (31%)
NF1 with low grade glioma	21 (20%)
Low grade glioma	32 (31%)
Other brain tumors	15 (14%)
Other	4 (4%)
Progression of brain tumor	
No	98 (93%)
Yes	7 (7%)
Medication	
None	71 (68%)
Yes	34 (32%)
Medical Therapy	
Observance only	52 (50%)
Surgery only	22 (21%)
Surgery + chemotherapy	6 (6%)
Surgery + chemotherapy + radiation therapy	10 (9%)
Surgery + radiation therapy + chemotherapy + antiangiogenic therapy	2 (2%)
chemotherapy only	10 (9%)
chemotherapy + radiation therapy	2 (2%)
none	1 (1%)

**Table 2:** Neuropsychological tests.

Neuropsychological Construct	Name of Test	Publication Year
Intelligence	Wechsler Intelligence Scale for Children—Fourth Edition [34]	2011
	Wechsler Preschool and Primary Scale of Intelligence—Third Edition[35]	2014
	Adaptives Intelligenzdiagnostikum III [36]	2014
	Bayley Scales of Infant and Toddler Development-Third Edition [37]	2015
Attention	Kinderversion der Testbatterie zur Aufmerksamkeitsprüfung (KITAP) [38]	
	Konzentrations-Handlungsverfahren für Vorschulkinder [39]	2006
Memory	Verbaler Lern- und Merkfähigkeits Test [40]	2001
	Wiener Entwicklungstest [41]	2012
	Developmental Scoring System for the Rey Osterrith Complex Figure [42]	
Behavior	Strengths and Difficulties Questionnaire [43]	1999

**Table 3:** Comparisons of the groups MRI success vs. no MRI success regarding neuropsychological and behavioral variables. MRI success was used as a dichotomous grouping variable, while neuropsychological and behavioral variables were used as outcomes. Negative Cohen’s *d*s indicate lower values in the no-success group compared to the success group.

**Psychometric Assessment**	***t (df)***	***p***	**Cohen’s *d***	
IQ (Wechsler test)	0.06 (28.67)	0.95	0.02	
IQ (AID-3)	−1.38 (13.67)	0.19	−0.57	
Range of intelligence (AID-3)	0.18 (10.36)	0.86	0.08	
Memory (VLMT/WET)	−3.03 (38.28)	0.00 *	−0.72	
KHV-VK: time	0.27 (23.12)	0.79	0.09	
KHV-VK: errors	−0.71 (23.34)	0.48	−0.25	
KiTAP: time in distractibility condition	0.37 (21.18)	0.72	0.11	
KiTAP: correctness (distractibility condition)	−0.10 (13.08)	0.92	−0.04	
KiTAP: completeness (distractibility condition)	−0.94 (21.06)	0.36	−0.29	
**SDQ Category (Normal vs. Critical Range)**	***χ*^2^ (*df*)**	***p***	***OR***	**Cramér’s *V***
Overall stress	2.57 (1)	0.11	2.37	0.19
Emotional distress	0.42 (1)	0.52	1.44	0.08
Behavioral difficulties	0.74 (1)	0.39	1.59	0.10
Hyperactivity and attentional difficulties	8.31 (1)	0.00 *	4.73 *	0.34
Difficulties getting along with others	0.00 (1)	0.97	1.02	0.00
Kind and helpful behavior	1.52 (1)	0.22	2.11	0.14

* *p* < 0.05.

## Data Availability

The datasets generated and/or analyzed during the current study are available from the corresponding author on reasonable request.
